# Effects of 16% Carbamide Peroxide Bleaching on the Surface Properties of Glazed Glassy Matrix Ceramics

**DOI:** 10.1155/2020/1864298

**Published:** 2020-02-10

**Authors:** Necla Demir, Muhammet Karci, Mutlu Ozcan

**Affiliations:** ^1^Selcuk University, Faculty of Dentistry, Department of Prosthodontics, Konya, Turkey; ^2^Alanya Oral Health and Dental Center Clinic, Alanya, Turkey; ^3^Clinic for Fixed and Removable Prosthodontics and Dental Materials Science, Center for Dental and Oral Medicine, University of Zurich, Zurich, Switzerland

## Abstract

**Objective:**

To determine the influence of the home bleaching agent, Opalescence PF, on the surface roughness and microhardness of glazed glassy matrix CAD-CAM ceramics. *Materials and Methods*. The 28 sintered leucite- and lithium disilicate-reinforced ceramic specimens (IPS Empress CAD and IPS e.max CAD) were divided into control and bleached groups. The home bleaching agent was applied to specimens of bleached groups for 7 days. The surface roughness and microhardness of all specimens were measured. A scanning electron microscope was used to evaluate the surface properties. The data were statistically analyzed by two-way ANOVA.

**Results:**

The control e.max CAD showed the lowest surface roughness values. For both Empress and e.max CAD, surface roughness was significantly higher for the bleached group (*p* < 0.05). No significant differences in microhardness were observed.

**Conclusions:**

According to our study, patients should be careful when using home bleaching agents because whitening agents can affect the mechanical properties of full ceramic restorations like e.max CAD and Empress CAD. Ceramic polishing may be required in clinical situations where ceramic restorations are accidentally exposed to bleaching gels.

## 1. Introduction

The esthetic smile of a patient is mainly affected by the color, shape, and position of the teeth [1]. The increase in demand from patients for a more esthetically pleasing smile has played an important role in esthetic dental materials being preferred for restorations [2]. Nowadays, full ceramic restorations are commonly available through the use of computer-aided design-computer-aided manufacturing (CAD-CAM) technology [3], with this becoming popular due to its excellent mechanical properties [4]. In particular, leucite-reinforced glass ceramic and lithium disilicate-reinforced glass ceramics have been preferable options for all-ceramic restorations because of their advantages like good mechanical resistance, translucency, and acid sensitivity. They are routinely used for manufacturing of crowns, inlays, onlays, and veneer restorations [5, 6].

The roughness of intraoral hard surfaces enhances initial adhesion and retention of oral microorganisms and accelerates maturation of plaque through increasing the area available for adhesion by a factor of 2 to 3. A rough surface may as well abrade opposing tooth or restorative materials. Thus, for optimum esthetics, the surface of dental restorations should be as smooth as possible [7, 8].

Bleaching techniques can be classified according to whether the bleaching is performed in the office or has an at-home component or both. The 30% to 35% hydrogen peroxide or carbamide peroxide are used for office bleaching for 15–60 minutes duration, whereas 10% to 16% carbamide peroxide (CP) can be used for home bleaching within a 1–4 week bleaching period with an application time of 4–8 hours each day [9]. The most efficient and safe bleaching technique is the one applied at home because it reduces the chance of side effects. This is because dentists are suspicious about the wide use of dental bleaching treatment and the possible effects hydrogen peroxide can have on dental ceramics. Bleaching materials and methods may have varying effects on restorative materials [10–14]. Especially, when bleaching has not been applied by the patient under the supervision of a dentist, misapplication of the bleaching agent may occur, leading to prosthetic restorations. Chemical softening of the restorative materials caused by these bleaching agents may affect their microhardness and surface roughness and, therefore, the clinical longevity of tooth-colored restorations [1, 15]. Decreases [13, 16] and increases [13, 17, 18] in microhardness have been detected for different types of bleaching methods, but no significant alterations have been pointed out [19–21]. The bleaching procedure results in a possible increase in plaque accumulation and affects the esthetics by changing the texture of the glazed ceramic restoration.

With this background, the aim of the present study was to evaluate the effect of 16% CP bleaching agent on the surface roughness and microhardness of glazed leucite- and lithium disilicate-reinforced ceramics (e.max CAD and Empress CAD). The null hypothesis of the present study was that Opalescence PF would not change the surface roughness and microhardness of glazed CAD-CAM ceramic systems.

## 2. Materials and Methods

A power analysis was performed (G^*∗*^ Power software v.3.1.10) to calculate the sample size required for four groups (Empress CAD‐Control, Empress CAD‐16% CP, e.max CAD‐Control, e.max CAD‐16% CP). The results indicated an actual power value of 94 for an effect size of *f* = 0.8, *α* = 0.05, noncentrality parameter of 18, and critical *t* value of 2.8. A requirement of 7 specimens in each group was determined. Opalescence PF (Ultradent, S Jordan UT, USA), a commonly available home bleaching system containing 16% CP, was used in this study. The CAD-CAM restorative materials used for this study included leucite-reinforced glass ceramic (Empress CAD; Ivoclar Vivadent, Schaan, Liechtenstein) and lithium disilicate-reinforced glass ceramic (e.max CAD; Ivoclar Vivadent, Schaan, Liechtenstein). The materials, their contents, and manufacturers are listed in Table 1.

Blocks of Empress CAD were cut using a diamond saw (Isomet 1000, Buehler, Lake Bluff, IL, USA), and the blocks of green stage e.max CAD were heated according to the manufacturer's specifications (845°C for 10 min) and then cut. The 28 sintered ceramic specimens, of length 15 mm, width 10 mm, and thickness 1 mm, were divided into four groups according to the ceramic type and surface treatment (*n* = 7). These four groups were as follows:

  Group 1: IPS Empress CAD (control group-just glazed)
  Group 2: IPS Empress CAD (glazed and treated with 16% CP)
  Group 3: IPS e.max CAD (control group-just glazed)
  Group 4: IPS e.max CAD (glazed and treated with 16% CP)

These specimens were polished using a 600-800-1200-2500-grit silicon carbide paper (Buehler, Lake Bluff, IL, USA) and then ultrasonically cleaned in distilled water for 5 min. Thereafter, all porcelain specimens were glazed in accordance with the manufacturer's instructions. Subsequently, all specimens were stored in distilled water at 37°C for 24 h. Finally, the thickness of each specimen was measured using a digital micrometer to ensure a final thickness of 1 mm (Mitutoyo IP65, Kawasaki, Japan).

Following this preparation, a thin layer of the bleaching agent (Opalescence PF gel) was applied to the surface of the specimens in Group 2 and 4 using an applicator, by the same clinician, at room temperature (according to the manufacturer's instructions) and then stored at 37°C during the bleaching period. Opalescence PF gel (Ultradent, S Jordan UT, USA) was left on the specimens for 6 h per day for 7 days. At the end of each bleaching exposure, the treated specimens were washed under running distilled water for 1 min and placed in fresh distilled water at 37°C until the next application in order to simulate the clinical situation between each bleaching treatment. The specimens of control groups (Groups 1 and 3) were placed in distilled water at 37°C for 7 days.

For each restorative material, the microhardness of the specimens was measured using a digital microhardness tester (Vickers Hardness Testing Machine; Shimadzu). Simultaneously, microhardness measurements for each ceramic sample were made on the ground surfaces using a hardness indentation device (force of 1.96 N for 15 s). The six measurements were made in two parallel lines of three measurements each, with the two lines located at a distance of 1 mm from the two opposing edges. Programming of the hardness indentation device and reproducible placement of the sample ensured that the indentations were made in exactly the same position on every sample. Surface roughness was measured using a single blinded evaluator for bleached and control groups. A prophylometer (Mitutoyo SJ-201, Kawasaki, Japan), featuring a microneedle, was utilized to scan the specimen surfaces to determine the average surface roughness (Ra). Three points were initially marked to ensure repeatable measurements. From these points, three parallel measurements in a longitudinal direction were performed on each specimen surface, with a 0.8 mm cutoff (*λ*c), at 0.5 mm/s. The number of sampling lengths was set to 5. The surface roughness was recorded for each specimen and a mean roughness (Ra, expressed in *µ*m) for each sample.

Representative scanning electron microscopy (SEM; EVO LS10; Zeiss, Cambridge, United Kingdom) images, obtained at ×10k magnification, were obtained for each group, showing their surface morphology. The specimens were dried, sputter-coated with gold, and one sample from each surface-treated and control group was examined to determine the morphologic effects on glazed surfaces of ceramics.

### 2.1. Statistical Analysis

The data retrieved were analyzed using IBM SPSS Version 23 (SPSS INC, Chicago, IL, USA). The Shapiro–Wilk test was used to verify a normal distribution of the data. Following this, the microhardness and surface hardness values were analyzed using the two-way ANOVA test to evaluate the differences between the groups (*α* = 0.05).

## 3. Results

**Figure 1:**
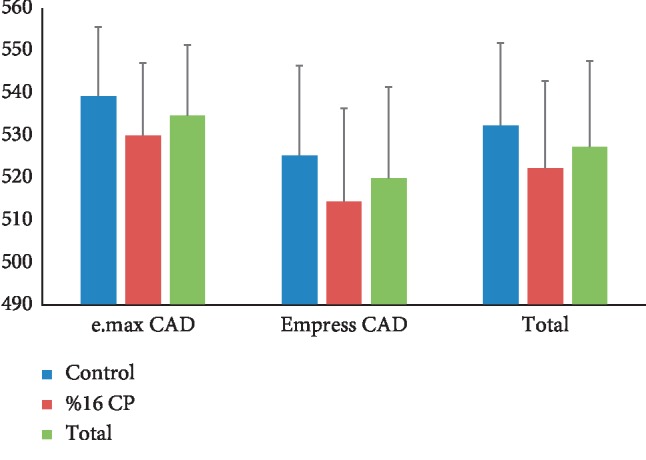
Mean and standard deviation values of microhardness of specimens.

No significant differences in microhardness values of glazed ceramic surfaces were observed between the control group and bleached groups according to the two-way ANOVA test. (Table 2) However, the order of ceramic systems in terms of the mean value of microhardness was e.max CAD (control) > 16% CP-treated e.max CAD > Empress CAD (control) > 16% CP-treated Empress CAD (Table 3). The microhardness of 16% CP-treated e.max CAD was higher than 16% CP-treated Empress CAD. The mean microhardness values of control and bleaching groups for Empress CAD were lower than E-max CAD ceramic. Furthermore, the reduction in the mean microhardness values for 16% CP-treated e-max CAD ceramic was less than that for 16% CP-treated Empress CAD ceramic (Figure 1).

**Figure 2:**
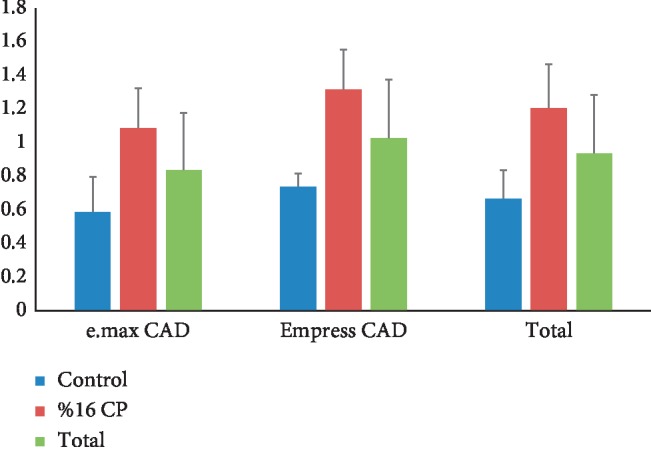
Mean and standard deviation values of surface roughness of specimens.

On the contrary, statistically significant differences were observed between the groups (*p* < 0.05) with regard to their surface roughness values (Table 4). The control glazed e.max CAD specimens showed the lowest surface roughness values (Figure 2). For both Empress and e.max CAD samples, surface roughness of glazed ceramic surfaces was significantly higher for the bleached group. (Table 5). Specifically, the order of the ceramic systems, in terms of mean value of surface roughness, was 16% CP-treated Empress CAD >16% CP-treated e.max CAD > Empress CAD (control) > e.max CAD (control). The mean of total surface roughness values of Empress CAD and bleached groups was significantly higher than that of E-max CAD ceramic and control groups (Table 5).

### 3.1. SEM Analysis

**Figure 3:**
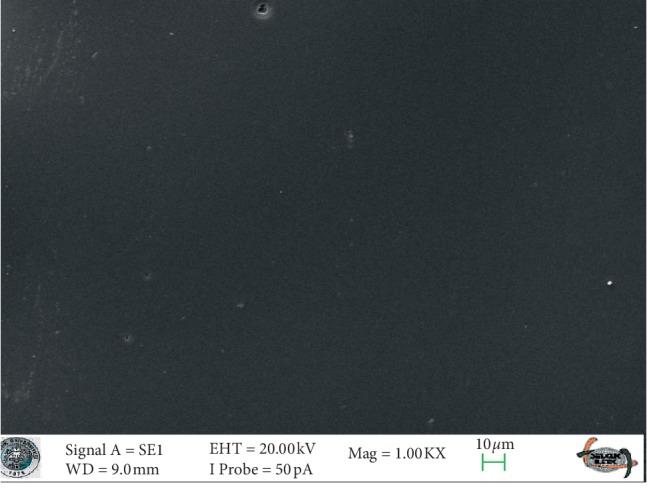
The SEM micrograph of the control group of the Empress ceramic system.

**Figure 4:**
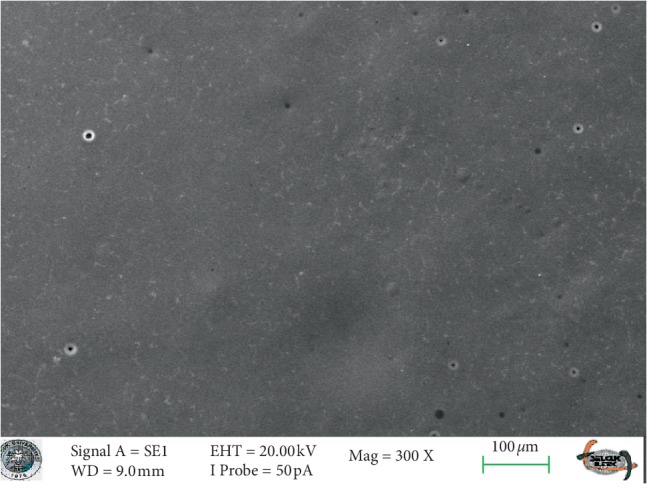
The SEM micrograph of the control group of the e-max ceramic system.

**Figure 5:**
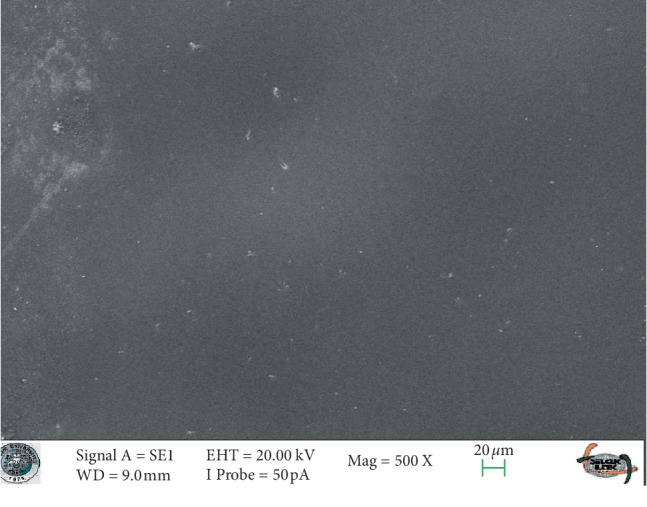
The SEM micrograph of the bleached group of the Empress ceramic system.

**Figure 6:**
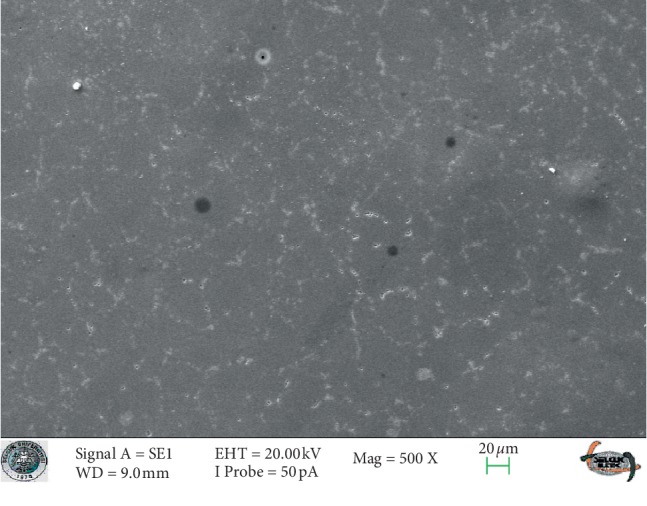
The SEM micrograph of the bleached group of the e-max ceramic system.

SEM images of glazed Empress CAD and e.max CAD ceramics (control groups) revealed smooth surfaces (Figures 3 and 4). The SEM micrographs of the glazed surfaces of feldspathic porcelain specimens appeared different from those of the control groups (Figure 5 and 6). These micrographs of bleached groups showed higher surface porosity and cracking in some areas (Figure 5 and 6). However, the control specimens showed some indentations due to the polishing procedures. The control e.max CAD specimens also showed the smoothest surface after bleaching, according to the SEM micrographs. Additionally, 16% CP-treated Empress CAD appeared more porous than 16% CP-treated e.max CAD ceramics, which is compatible with the statistical results.

## 4. Discussion

The null hypothesis of the study for microhardness testing was accepted; however, the null hypothesis of the study for the surface roughness testing was rejected. There are many studies discussing the influence of bleaching agents on the surface properties of restorative materials and dental tissues. On the other hand, little is known about the influence of bleaching on ceramics. It was reported that minor surface alterations, as determined by SEM studies, and decrease in surface microhardness and fracture strength may occur as a result of bleaching dental hard tissues [22–25]. Besides these effects of bleaching agents on dental hard tissue, some clinicians worry about the influence of these agents on dental ceramic materials [26, 27]. The bleaching material may change the structural and mechanical properties of the restorative material, leading to failures [28]. The high surface roughness, normally increased through finishing and polishing, needs to be reduced because surface roughness greatly effects esthetical, biological, and mechanical properties of ceramic restorations. The increase in roughness of restoration surfaces can cause increased discoloration [1]_,_ may simplify plaque aggregation [29], and can also cause abrasion and increased wear of antagonists [30, 31]. Finally, high surface roughness has usually been found to negatively affect porcelain durability [32, 33].

A glazed ceramic surface is generally considered favorable, as it is thought to increase the fracture resistance and reduce the potential abrasiveness of the ceramic surface by sealing the open pores on the surface of the fired porcelain [34]. The specimens were also glazed to simulate a clinical scenario. Furthermore, the glazing process reduces porosity on the surface of the ceramic material and the lower the roughness on such surfaces, the lower the risk of micro-organism colonization, e.g., *Candida albicans* from the intraoral environment [35]. CP is the most commonly used home bleaching agent, so we used it in our study. Although dental ceramics are the most biocompatible among all dental restorative materials, their surfaces can show surface disruption comparable with acidulated fluoride gels or other solutions [36]. The reaction of CP releases hydrogen peroxide and free radicals, which are in charge of dental bleaching [37, 38]. During this process, the contact and possible diffusion of free radicals of H^+^ or H_3_O^+^, produced by bleaching agents, may selectively leach alkali ions and, subsequently, cause dissolution in ceramic glass networks. This causes extended exposure to CP and may harm the dental porcelain and may alter the surface properties of the porcelain surface. The mechanism of how bleaching regimens affect restorative material is not clear, but presumably, this may be due to break down of CP into hydrogen peroxide and urea in an aqueous solution, with hydrogen peroxide being the active bleaching agent, which may penetrate the surface of restorative materials [16].

The effect of the bleaching agent is related to the depth of its penetration into the restorative materials [39]. Today, this bleaching technique may be performed at home for 1–8 h a day according to manufacturers' instructions. In order to simulate this accurately, herein we applied Opalescence PF to the ceramics for 6 h per day for 7 days.

Anusavice et al. reported that ceramics should be chemically stable in the mouth, because dental prostheses must withstand degradation [36]. Otherwise, ceramics could release potentially toxic substances and radioactive components, exhibiting increased wear, abrasion of opposing dental structures, and increased plaque adhesion because of exposure to such intraoral challenges [36]. Zaki et al. and Bollen et al. found that increase in surface roughness beyond the threshold of Ra = 0.2 *µ*m, as in this study, may enhance plaque accumulation, thereby increasing the risk of both secondary caries and periodontal inflammation and affecting ceramic esthetics by changing the ceramic texture [40, 41]. Zaki et al. [40] also found that bleaching significantly increased the roughness of the polished overglazed ceramic as we found higher surface roughness values for our bleached overglazed ceramics in our study. This higher roughness values may have been caused by etching of the ceramic caused by the carbamide peroxide agent. This ﬁnding also agrees with that of White et al. [42], Rosentritt et al. [12], and Silva et al. [43]; however, Duschner et al. [44] reported no changes in surface morphology of porcelain exposed to bleaching. This could have been due to the lower concentration of the bleaching agents in their study. Our results do not either corroborate with those of Anusavice et al. [36] and Zavanelli et al. [45], who found no alterations on ceramic surfaces treated separately with 10% and 15% carbamide peroxide for 126 h. However, other authors [43, 46, 47] have demonstrated that bleaching gels affected the surface roughness of dental ceramics, as we found in our study. According to these authors, these results were related to the leaching of components from the porcelain matrix as a function of continuing peroxide application [43, 46, 47].

Butler et al. [48] reported that porcelains might have significant roughening from 10% CP treatment as we found in our study for 16% CP treatment. The outcomes of Butler's study reveal that the feldspathic porcelain showed a significantly rougher surface after 21 days of exposure to both 10% and 35% CP agents (*p* < 0.05). We speculate that this result is related to a leach of any component from glazed porcelain matrix as a function of continual peroxide application. Turker and Biskin [49] also recorded that the surface roughness of overglazed bleached ceramic samples increased signiﬁcantly during the ﬁrst two weeks as we found higher surface roughness values for bleached groups in our study for the first week.

It is known that hardness is related to a materials' strength, proportional limit, and its ability to abrade or to be abraded by opposing dental structures' materials [49]. Therefore any chemical softening resulting from bleaching might have implications on the durability of restorations. In the current study, no surface microhardness changes were observed in all tested 10% CP groups. Turker et al. [49] also reported that using 10% CP or 16% CP did not affect the microhardness of the restorative materials as we found in our study.

Bahannan [50] found that the microhardness of feldspathic ceramic was not affected by different concentrations of CP. Bahannan also found that there were no significant differences in the microhardness of feldspathic porcelain (10% CP) as we found in our study. The results in this study are also compatible with those of Zavanelli et al. [45], who found no microhardness alterations on ceramic surfaces treated with 10% or 15% CP for 126 h. Furthermore, this study depicted that no difference in ceramic surface hardness was observed for bleached and control groups. Polydorou et al. [51] found that the microhardness of the ceramic was not affected by the bleaching agents as it was in our study.

The limitation is that an energy-dispersive X-ray microanalysis of ceramic surfaces was not determined [1]. On the other hand, saliva and masticating forces are important factors during and after bleaching. They may affect the mechanical response of the materials. The lack of these forces may be another limitation in our study, but can be investigated in the future by in vivo studies.

According to Attin et al. [9], none of the studies mentioned above investigated how much the induced porosities increased the surface roughness of the tested glazed material, such that it led to the need for replacement of existing restorations after bleaching, in order to ensure longevity of the restorations. Therefore, it remains speculative whether these changes of surface texture and hardness are relevant under clinical conditions or if they are barely a surface phenomenon that could be removed by simple polishing of restorations. However, polishing of the restorations after bleaching is advisable at least. As assessed by Mor et al. [52], this is because the increased surface roughness is held responsible for increased adherence of certain cariogenic microorganisms on the outer surface of tooth-colored restorative material after contact with different bleaching agents.

## 5. Conclusion

Within the limitations of this study, it can be concluded that

1. High-concentration CP at-home bleaching agents significantly affect the surface roughness of dental ceramics, so ceramic restorations should be protected before any bleaching for fear of roughness.
2. Patients who have full ceramic restorations such as e.max CAD and Empress CAD should be careful while applying the home bleaching treatment. There may be the need for ceramic polishing in clinical situations where ceramic restorations are accidentally exposed to bleaching gels.
3. The small insignificant microhardness changes could lead to further alterations like discoloring of the materials. Further clinical research is necessary.

## Figures and Tables

**Table 1:** Ceramics and bleaching agent.

Materials	Manufacturer	Shade	Lot number
e.max CAD	IvoclarVivadent	**LT-A2**	**T27280**
Empress CAD	IvoclarVivadent	**LT-A2**	**N79016**
Opalescence PF %16 (carbopol, pH = 6)	Ultradent Products, Inc.		**BBHY1**

**Table 2:** The two-way analysis of variance for microhardness testing.

Source	Type III sum of squares	df	Mean square	*F*	*p*
Ceramic	1564,518	1	1564,518	4,136	0,053
Surface treatment	727,260	1	727,260	1,922	0,178
Ceramic *∗* surface treatment	4,560	1	4,560	0,012	0,913

**Table 3:** Mean (SD) values of microhardness testing.

	e.max CAD	Empress CAD	Total
Control	539,7 (16,4)	525,6 (21,3)	532,7 (19,7)
%16 CP	530,3 (17,3)	514,6 (22,2)	522,5 (20,8)
Total	535,0 (16,9)	520,1 (21,7)	527,6 (20,5)

**Table 4:** Two-way analysis of variance for surface roughness testing.

Source	Type III sum of squares	df	Mean square	*F*	*p*
Ceramic	0,241	1	0,241	5,699	**0,025** ^*∗*^
Surface treatment	2,041	1	2,041	48,179	**<0,001** ^*∗*^
Ceramic *∗* surface treatment	0,010	1	0,010	0,246	0,625

^*∗*^ *p* < 0.05 represents significant difference.

**Table 5:** Mean (SD) values of surface roughness testing.

	e.max CAD	Empress CAD	Total
Control	0,59 (0,21)	0,74 (0,08)	0,67 (0,17)^*∗*^
%16 CP	1,09 (0,24)	1,32 (0,24)	1,21 (0,26)^*∗*^
Total	0,84 (0,34)^*∗*^	1,03 (0,35)^*∗*^	0,94 (0,35)

^*∗*^ *p* < 0.05 represents significant difference.

## Data Availability

No data were used to support this study.

## Additional Points

Bleaching can affect the surface properties of glazed glassy matrix ceramics. There may be the need for ceramic polishing in clinical situations where ceramic restorations are accidentally exposed to bleaching gels.
